# A Private Hospital for Austin

**Published:** 1892-01

**Authors:** 


### A PRIVATE HOSPITAL FOR AUSTIN.

Drs. Wooten, McLaughlin, Bennett and Tyner, of Austin, have petitioned the City Council, to lease the City aad County Hospital for a term of years. They offer for the sum of $3,600 a year, to give the city the same service, *i. e.*, to care for and treat in hospital the same average number of patients which the official records show to have been cared for for the last four years, except that the service will be of a better quality, and with all the adjuncts of trained nurses, modern methods of hygiene, of treatment, etc. This sum, $3,600, is $803 a year less than the average annual cost to the city of conducting the hospital. All excess of patients over the average as shown by the records, the petitioners propose to care for at $1.00 per capita per day. This is also much less than the average cost per capita for the four years.

It is the aim of these gentlemen, should they secure the hospital, to convert it into a private infirmary, where, in addition to caring for the indigent of the city, they can receive and treat their patients, medical surgical and gyneological.

This is something badly needed in Austin, and the JOURNAL indulges the hope that the city will see it to her interest financially, to lease the hospital to the petitioners; for there is no place in Austin where a patient, be he ever so wealthy, can be comfortably cared for during sickness; not even the best hotel affords such facilities as doctor and patient should have. In brief, Austin needs a private hospital, and needs it badly; and these gentlemen should have the backing of the entire community in their efforts to establish one.

